# Feasibility, safety, acceptability, and functional outcomes of playing Nintendo Wii Fit Plus™ for frail elderly: study protocol for a feasibility trial

**DOI:** 10.1186/s40814-017-0184-1

**Published:** 2017-10-24

**Authors:** Gisele Cristine Vieira Gomes, Jéssica Maria Ribeiro Bacha, Maria do Socorro Simões, Sumika Mori Lin, Larissa Alamino Pereira Viveiro, Eliana Maria Varise, Wilson Jacob Filho, José Eduardo Pompeu

**Affiliations:** 1 Department of Physical Therapy, Speech Therapy, and Occupational Therapy, School of Medicine, University of Sao Paulo, 51th Cipotânea Street, University City, Sao Paulo, 05360-000 Brazil; 2 Department of Frail Syndrome, School of Medicine, University of Sao Paulo, 255, Doctor Olívio Pires Campos Street, Sao Paulo, 05403-000 Brazil; 3 Department of Neuroscience and Behavior, University of Sao Paulo, 1721, Professor Mello de Morais Avenue, University City, Sao Paulo, 05508-030 Brazil

**Keywords:** Aged, Frail elderly, Virtual reality therapy, Rehabilitation

## Abstract

**Background:**

Frailty can be defined as a medical syndrome with multiple causes and contributors, characterized by diminished strength and endurance and reduced physiological function that increases the vulnerability to develop functional dependency and/or death. Studies have shown that the most commonly studied exercise protocol for frail older adults is the multimodal training. Interactive video games (IVGs) involve tasks in virtual environments that combine physical and cognitive demands in an attractive and challenging way. The aim of this study will be to evaluate the feasibility, safety, acceptability, and functional outcomes of playing Nintendo Wii Fit Plus^TM^ (NWFP) for frail older adults.

**Methods/design:**

The study is a randomized controlled, parallel group, feasibility trial. Participants will be randomly assigned to the experimental group (EG) and control group (CG). The EG will participate in 14 training sessions, each lasting 50 min, twice a week. In each training session, the participants will play five games, with three attempts at each game. The first attempt will be performed with the assistance of a physical therapist to correct the movements and posture of the patients and subsequent attempts will be performed independently. Scores achieved in the games will be recorded. The participants will be evaluated by a blinded physical therapist at three moments: before and after intervention and 30 days after the end of the intervention (follow-up). We will assess the feasibility, acceptability, safety, and clinical outcomes (postural control, gait, cognition, quality of life, mood, and fear of falling).

**Discussion:**

Due to the deficiencies in multiple systems, studies have shown that multimodal interventions including motor-cognitive stimulation can improve the mobility of frail elderly adults. IVGs, among them the NWFP, are considered as a multimodal motor-cognitive intervention that can potentially improve motor and cognitive functions in the frail elderly. However, there is still no evidence in the literature that proves the feasibility, safety, acceptability, and functional outcomes of this intervention in frail elderly individuals.

**Trial registration:**

Brazilian Registry of Clinical Trials (RBR-823rst). World Health Organization Trial Registration Data Set (Additional file 1).

**Electronic supplementary material:**

The online version of this article (10.1186/s40814-017-0184-1) contains supplementary material, which is available to authorized users.

## Background

Frailty can be defined as a medical syndrome with multiple causes and contributors, characterized by diminished strength and endurance and reduced physiological function that increases an individual’s vulnerability to develop increased dependency and/or death [1]. A recent systematic review [2], incorporating 31 studies of frailty in persons aged 65 years or older, found a prevalence of from 4.0 to 17.0% (mean 9.9%) of physical frailty, with a higher prevalence when psychosocial frailty was also included. Women (9.6%) were almost twice as likely as men (5.2%) to be frail [1]. The prevalence of frailty is markedly increased in persons older than 80 [1].

Frail older adults have a high risk of becoming dependent; however, with adequate intervention (mainly nutrition and physical exercise), frailty can be reversed to a robust state [3]. A systematic review [4] showed that the best strategy to reduce the number of falls, improve gait and balance, and increase muscle strength in frail elderly individuals is interventions based on combined exercises, such as resistance training, muscle strengthening, flexibility, balance and coordination training, and aerobic exercises.

New types of intervention have been proposed as complementary tools for rehabilitation of the elderly, among them interactive video games (IVGs) that can optimize motor learning and neural plasticity [5]. IVGs combine motor and cognitive tasks which are performed in a motivating and challenging virtual environment [6]. IVGs stimulate complex and dynamic movements that are similar to the movements required in daily life [5]. In addition, IVGs are inexpensive, fun, and can be used by patients who live in remote settings. Together, these factors can help to improve adhesion to and results of rehabilitation [6].

Among IVGs, Nintendo Wii Fit Plus® (NWFP) games have been considered as an inexpensive motor-cognitive intervention. The games are played using the Balance Board, a wireless platform that detects body oscillation through four sensors that identify the center of pressure of the player. A recent systematic review [7] investigated the effects of IVGs on balance of different populations, among them, elderly people. Twelve of the selected studies assessed the effects of the NWFP and showed that the NWFP was effective for improving balance [8–10].

We performed an extensive search in the PubMed, Web of Science, and PEDro databases and found only one pilot study [11] which compared the effectiveness of the NWFP to improve mobility of pre-frail elderly people. The participants were randomized to one of three groups: (1) control, (2) seated exercise (traditional senior fitness program), or (3) NWFP group (basic games and addition of weight vest with 2% of their body weight every 2 weeks). The training of groups 2 and 3 was performed for 45 min, three times a week, for 15 weeks. There was improvement in physical functional status in the seated exercise and NWFP group compared to the control group. Despite the positive effects of the NWFP group, there were some methodological issues in this pilot study such as the absence of blindness of the subjects, therapists, and evaluators and the absence of the intention-to-treat analysis, since there was a dropout of 12 participants during the study. Furthermore, this pilot study did not assess the feasibility, safety, or acceptability of the NWFP for pre-frail elderly people. Finally, the participants of the NWFP group performed their training using a weight vest. The addition of weight certainly interfered in the intensity of the intervention with the games. In fact, the weight addition makes it difficult to infer whether the improvement occurred due to training with the games or the resistance of the weight vest.

Despite evidence of the positive effects of IVGs on clinical outcomes of community dwelling elderly people [12–20], it is not clear whether IVGs are feasible, safe, and acceptable for frail elderly people. Furthermore, the effectiveness of IVGs on this population has not been established.

### Objectives

The current study aims to evaluate the feasibility, safety, acceptability, and functional outcomes of playing NWFP in frail older adults.

### Hypotheses

1. We speculate that the frail elderly adults will improve their performance in the games and increase their scores;
2. We hypothesize that the intervention based on the NWFP games will not cause any adverse events such as syncope, dizziness, vertigo, falls, or any other medical condition that requires hospitalization or leads to disability. A previous study [24] showed that elderly individuals with Parkinson’s disease presented good acceptability to training with the NWFP;
3. We expect that the NWFP games will be acceptable to frail elderly adults. Despite this population possibly not being familiar with this kind of technology, the NWFP games were developed for entertainment with attractive and motivating virtual environment and are easy to use. A previous study [12] showed that elderly adults demonstrated good acceptability to training with NWFP;
4. The training sessions with the NWFP will provide improvement in postural control, gait, quality of life, cognition, and mood.

### Trial design

The study will be a randomized controlled, parallel group, feasibility trial. Participants will be randomly assigned 1:1 to NWFP training or usual care. Study assessments will be conducted before and after the intervention and 30 days after the end of the intervention (follow-up).

## Methods

### Study setting

All participants will be recruited from the Frailty Ambulatory Clinics at the Hospital of the Medical School, University of Sao Paulo, Brazil.

### Eligibility criteria

#### Inclusion criteria

- Pre-frail and frail older adults aged 60 years or older, diagnosed with frailty syndrome according to Fried’s criteria: slow walking speed, impaired grip strength, self-reports of declining activity levels, exhaustion, and unintended weight loss (pre-frail—presence of at least one deficit and frail—presence of at least three deficits) [21];
- Capacity to maintain a standing position and walk independently;
- Normal or corrected visual acuity evaluated by the Snellen Scale [22];
- Good hearing acuity, clinically assessed by the whisper test [23] without previous experience with the NWFP;
- Agreement to participate by signing the informed consent form.

### Exclusion criteria

- Participants presenting with clinical conditions that could preclude the performance of physical exercises in an orthostatic position, such as cardiovascular, orthopedic, or neurological conditions, and older adults unable to interact with the games.

### Administration of assessments

The same-trained researcher will provide the patient with a free informed consent form, which also contains information on the possible benefits that the interventions may bring and possible risks and indemnities to patients, also emphasizing that the patient can withdraw from the research at any time without any contradictions. On the same day, the evaluator will clarify all doubts regarding the research project and inform the volunteers that all data resulting from the survey will be used for scientific purposes. The same-trained researcher, blinded to treatment allocation, will evaluate all participants at three timepoints: immediately pre and post interventions and 30 days after the end of the interventions (follow-up). Participants will be asked not to inform the evaluators about the kind of intervention they have received.

### Intervention

Participants will be randomly assigned to the experimental group (EG) and control group (CG), with an allocation ratio of 1:1.

Participants in the EG will participate in 14 training sessions, lasting 50 min, with a frequency of twice a week. In each training session, the participants will play five games and will be allowed three attempts at each game. The first attempt will be performed with the help of a physical therapist to correct the movements and posture of the patients through manual steering and verbal controls, while the subsequent attempts will be performed independently for the analyses of motor learning. Scores achieved in the games will be recorded. The time required to exchange the game will be approximately 2 min, and the patients will sit in a chair during this period.

Participants in the CG will be guided through verbal instructions and an illustrative booklet of guidelines carried out according to the World Health Organization, Policy Department number 385, 2014. This booklet describes physical activity, its benefits and risks, and encourages the study participants to seek health units near their residence where free physical activities are offered.

### Game selection and description

The games were selected based on their motor and cognitive demands. Table 1 shows the description of the games [24].

**Table 1:** Description of the games

Game	Motor demands	Cognitive demands
Table Tilt (TT)	Multi directional and controlled center of mass displacements	Planning the motor response to achieve the goal and controlling the time to completion of the task
Rhythm Parade (RP)	Stationary marching associated with movements, with upper limb movements	Division of attention between performing lower and upper limb movements and reaching random targets with movements of one or both upper limbs
Obstacle Course (OC)	Stationary marching	Attention and planning the rapid decisions to accelerate or decelerate the march
Single Leg Extension (SLE)	Stationary control of the center of mass	Maintaining the attention and imitation of the movements of the virtual trainer
Tilty City (TC)	Lateral displacement of the center of mass associated with upper limb movements.	Planning movements to reach objects as well as division of attention between the movements of the center of mass and upper limbs
Basic Step (BS)	Ability to climb up and down steps quickly following the rhythm of the game, requiring balance control in a one-way position	Attention to the visual and auditory stimuli that guide the task
Penguim (PG)	Rapid displacements of the center of masses lateral-lateral with the feet immobile	Planning the movements in the target’s directions
Heading Soccer (HC)	Displacement of the lateral-lateral center of mass	Make a quick decision between going against or deflecting the target
Basic Rum (BR)	Fast stationary gear on the ground	Division of attention between the march and the task of memorizing objects that will be questioned at the end of the course
Torso Twist (TTW)	Stationary control of the center of mass while performing trunk rotations by moving the upper limbs, keeping the feet immobile	Maintain attention and imitate the movements of the virtual trainer

### Outcome measures

#### Feasibility, acceptability, and safety outcomes

Feasibility will be assessed by the participants’ performance in the games, measured by the score achieved. Increasing scores indicate that the participant is capable of not only playing but also improving his or her performance in the games. This is the main measure of game play that promotes motivation [24]. To determine the feasibility of the program, we will collect attendance records from each participant before and after training sessions. We will consider the exercise program to be feasible if we maintain > 50% attendance for all sessions and average > 80% attendance per session.

Acceptability will be assessed through a game satisfaction questionnaire [25]. The questionnaire consists of 18 questions, among them are participants’ perception of games (“What do you like about the games?”; “Which game do you like best?”; “Which game do you like least?”); more difficult games (“Which game did you find most difficult?”); easier games (“Which game did you find easiest?”); motivation (“Did you feel motivated to play the games?”); and discomfort during training (“Did you feel any discomfort playing the games?”).

Safety will be assessed by the proportion of participants who experienced intervention-related adverse events or any serious adverse event during the study period. An adverse event is defined as any untoward medical occurrence, such as convulsions, syncope, dizziness, vertigo, falls, or any other medical condition that requires hospitalization or leads to disability. The therapist will register the occurrence of any adverse events, and the participant’s blood pressure, oxygen pulse saturation, heart and respiratory rates, and self-perception of effort, measured by the BORG Scale [26], will be assessed before and after each training session. We will monitor the onset of any clinical conditions that could preclude the performance of physical exercises in an orthostatic position, such as cardiovascular, orthopedic, or neurological conditions.

#### Clinical outcomes

Clinical outcomes will be the following: (1) postural control, assessed by the Mini-Balance Evaluation Systems Test (Mini-BEST-Test) [27]; (2) gait, assessed by the Functional Gait Assessment (FGA) [28]; (3) cognition, assessed by the Montreal Cognitive Scale (MoCA) [29]; (4) quality of life, assessed by the Brazilian version of the Short Form 36 (SF-36) [30]; (5) mood, assessed by the Geriatric Depression Scale (GDS-15) [31]; and (6) fear of falling, assessed by the Falls Efficacy Scale (FES-I) [32].

Due to the absence of specific studies on minimal detectable change (MDC) in frailty, we will consider a change of 3.5 points in the Mini-BEST-Test [33] and 4.2 points in the FGA [34] as significant. The MDC of the other scales has not yet been established.

### Participant timeline

Table 2 illustrates the process of enrolling participants in the study, the intervention, and timing of assessments.

**Table 2:** Schedule of enrolment, interventions, and assessments

	Study period				
Timepoint	Day 1	Day 2	Days 3 to 16	Day 17	Day 47
Enrollment	X				
Eligibility screen	X				
Informed consent	X				
Allocation	X				
Interventions			X		
Assessments		X		X	X
Initial assessment (pre)		X			
Final assessment (post)				X	
Follow-up assessment					X

### Sample size

As this is a feasibility study, no formal sample size calculation was performed [35]. Instead, this study follows sample size recommendations for pilot randomized controlled trials [36] and aims to have at least 12 participants per group who provide full data. We aim to recruit 15 participants for each group (i.e., total sample size of 30) to compensate for a 20% dropout. This number of participants is deemed adequate to provide sufficient information on key feasibility issues such as recruitment and acceptability of the intervention.

### Recruitment

Potentially eligible participants will be identified by the clinical care team of Frailty Ambulatory Clinics at the Hospital of the Medical School, University of Sao Paulo. This team will undertake the initial approach, explaining how the study will be conducted. If the participant is willing to participate, a suitably qualified person will then provide verbal and written information about the study.

### Randomization

Participants will be randomly assigned to the EG and CG, with an allocation ratio of 1:1. Randomization schedule will be prepared from a computer-generated list of random numbers, by a researcher not involved in the trial. In order to guarantee the balance between the groups regarding the level of frailty, we will stratify pre-frail and frail participants in the randomization.

### Blinding

Blinding of trial participants and the intervention facilitator is not possible. All outcomes will be assessed by a researcher blinded to group allocation. Participants will be asked not to disclose their allocation to the physical therapist who will participate in the assessments.

### Data collection

The participants will be interviewed regarding sociodemographic characteristics (age, gender, educational level, marital status, and family income), health (number of Fried’s criteria, number of falls in the previous 12 months, number of chronic diseases, and number of medications currently in use), and clinical conditions (postural control and balance, gait, cognition, quality of life, mood, fear of falling, and incidence of falls).

### Data management and monitoring

All electronic identifiable information will be held on a secure, password-protected database, accessible only to the research sponsor. Paper forms with identifiable information will be held in secure, locked filing cabinets within a restricted area. Participants will be identified by a code number only. Direct access to source data/documents will be required for trial-related monitoring by authorized personnel only. Personal data collected during the trial will be handled and stored in accordance with the 1998 Data Protection Act. All paper and electronic data will be retained for at least 5 years after completion of the trial.

### Statistical analyses

A detailed analysis plan will be prepared before all the data has been collected. Analyses will be conducted in Dell™ Statistica (version 13.0) using the principles of intention-to-treat. Descriptive statistics will be used to characterize the groups at baseline and present the feasibility outcomes. Although determining differences in clinical outcomes between the two groups is not the primary purpose of this trial, comparisons will be undertaken to investigate the feasibility of studying these outcomes and calculate estimates for the likely effect sizes and 95% confidence intervals. The effect size will be calculated in order to determine whether change can be detected over time using these outcome measures and to determine the most appropriate primary outcome. The focus of the results will be on the estimates of the treatment effects rather than statistical significance, and as such, no hypothesis testing will be undertaken [37]. Differences between the two comparison groups will be presented in the form of an unadjusted mean difference for continuous outcomes with their associated 95% confidence intervals.

### Adverse events

An adverse event is defined as any untoward medical occurrence in a participant which does not necessarily have a causal relationship with this intervention. Any adverse events will be reviewed by the study team, and likely causality will be assessed and reported on a form.

### Auditing

We will institute a rigorous program of quality control. The research sponsor in conjunction with the trial coordinator will be responsible for ensuring adherence to the trial protocols at the trial sites. Quality assurance checks will be undertaken by the University of Sao Paulo to ensure integrity of randomization, study entry procedures, and data collection.

### Protocol amendments

Any modifications to the protocol which may impact on the conduct of the study, potential benefits to the patient, or may affect patient safety, including changes in study objectives, study design, patient population, sample sizes, study procedures, or significant administrative aspects will require a formal amendment to the protocol. Such amendments will be agreed upon by the Ethics Committee of the Medical School of the University of Sao Paulo, Brazil, and the Brazilian Registry of Clinical Trials prior to implementation and notified to the health authorities in accordance with local regulations.

### Dissemination policies

The dissemination will be to inform a wide range of local, national, and international audiences about the results and conclusions. It must, however, be remembered as part of this strategy that the current project is preliminary work aimed at informing a subsequent definitive clinical trial. We aim to publish our research in journals that cover the relevant medical specialties and with preference for those that deposit publications in open access databases to increase free dissemination. In addition, we aim to present this research at appropriate national and international conferences.

## Discussion

The current study is designed to evaluate the feasibility, safety, acceptability, and functional outcomes of playing NWFP for frail older adults. This feasibility study is in preparation for a main trial that will go ahead if all feasibility criteria and hypothesis are met.

Frailty affects functionality in different aspects, such as mobility, gait, balance, muscle strength, motor processing, cognition, nutrition, and physical activity [38]. Due to the deficiencies in multiple systems, studies have shown that multimodal interventions including motor-cognitive stimulation can improve the mobility of frail elderly adults [39]. IVGs, among them the NWFP, are considered as a multimodal motor-cognitive intervention that can potentially improve motor and cognitive functions in the frail elderly. The NWFP can promote physical activity and improve balance, mobility, gait speed, muscle strength, flexibility, and functional abilities in older adults [40]. However, there is still no evidence in the literature that proves the feasibility, safety, acceptability, and functional outcomes of this intervention in frail elderly adults. We speculate that due to the challenging stimulation to the postural control and cognitive systems, added to factors that can improve motor learning such as visual and auditory feedback and motivating environment, the NWFP will be feasible, safe, and acceptable and will promote clinical benefit in frail elderly adults.

### Trial status

To date, we have recruited about 50% of the participants.

## Additional files

Additional file 1: World Health Organization Trial Registration Data Set. (DOCX 14 kb)

Additional file 2: School of medicine of the University of Sao Paulo free and informed consent term. (DOCX 15 kb)

## Abbreviations

CG: Control group
EG: Experimental group
FES-I: Falls Efficacy Scale
FGA: Functional Gait Assessment
GDS-15: Geriatric Depression Scale
IVG: Interactive video games
MDC: Minimal detectable change
Mini-BEST-Test: Mini-Balance Evaluation Systems Test
MoCA: Montreal Cognitive Scale
NWFP: Nintendo Wii Fit Plus^TM^
SF-36: Brazilian version of the Short Form 36
SP: Sao Paulo
