# Supplementary material for: Feasibility, safety, acceptability, and functional outcomes of playing Nintendo Wii Fit Plus™ for frail elderly: study protocol for a feasibility trial
Source: Pilot Feasibility Stud. 2017 Oct 24;3:41. doi: 10.1186/s40814-017-0184-1 (PMC5654058; doi:10.1186/s40814-017-0184-1)
Supplement: Supplementary file 1 — World Health Organization Trial Registration Data Set. (DOCX 14 kb) [file 40814_2017_184_MOESM1_ESM.docx]

**Additional files**

**World Health Organization Trial Registration Data Set**

| **DATA CATEGORY** | **INFORMATION** |
| --- | --- |
| Primary registry and trial identifying number | Ethics Committee of the Medical School of the University of Sao Paulo (1.150.930) |
| Date of registration in primary registry | 03 March 2016 |
| Secondary identifying numbers | Brazilian Registry of Clinical Trials: RBR-823rst. |
| Source(s) of monetary or material support | Not applicable |
| Primary and Secondary sponsor | School of Medicine, University of São Paulo - Department of Speech Therapy, Physiotherapy and Occupational Therapy |
| Contact for public queries | G.C.V.G; J.E.P. Phones: +55 11 [3091-84](about:blank)24. E-mail: [gigicv.gomes@gmail.com](mailto:gigicv.gomes@gmail.com) or j.e.pompeu@usp.br |
| Contact for scientific queries | G.C.V.G; J.E.P. Phones: +55 11 [3091-84](about:blank)24. E-mail: [gigicv.gomes@gmail.com](mailto:gigicv.gomes@gmail.com) or j.e.pompeu@usp.br |
| Public title | Feasibility, safety, acceptability and functional outcomes of playing Nintendo Wii Fit Plus^TM^ for frail elderly: Study protocol |
| Scientific title | Feasibility, safety, acceptability and functional outcomes of playing Nintendo Wii Fit Plus^TM^ for frail elderly: Study protocol |
| Countries of recruitment | Brazil |
| Health condition(s) or problem(s) studied | Elderly with frailty syndrome |
| Intervention(s) | Participants will be randomly assigned to the experimental group (EG) and control group (CG). The EG will participate in 14 training sessions, lasting 50 minutes, with a frequency of twice a week. The participants will be evaluated by a blinded physical therapist in three stages: before, after intervention, and 30 days after the end of the intervention (follow-up). The outcomes are: feasibility, acceptability, safety and clinical (postural control, gait, cognition, quality of life, mood, and fear of falling. |
| Key inclusion and exclusion criteria | **Inclusion Criteria:**  Pre-frail and frail older adults aged 60 years or older diagnosed with frailty syndrome according to Fried’s criteria. The five deficits of Fried’s criteria are: slow walking speed, impaired grip strength, self-reports of declining activity levels, exhaustion, and unintended weight loss (pre-frail - the presence of at least one deficit and frail - the presence of at least three deficits); capacity to maintain a standing position and walk independently; normal or corrected visual acuity evaluated by the Snellen Scale; good hearing acuity, clinically assessed by the whisper test, without previous experience with the NWFP, and agreement to participate by signing the informed consent form.  **Exclusion Criteria**  Participants who, during the period of the study, present any clinical changes that could preclude the performance of physical exercises in an orthostatic position, such as cardiovascular, orthopaedic, or neurological conditions, and older adults unable to interact with the games. |
| Study type | Randomised controlled feasibility trial  Interventional  Allocation: randomised  Intervention model: parallel assignment  Masking: blind (outcomes assessor) |
| Date of first enrolment | 03 March 2016 |
| Target sample size | 30 elderly individuals |
| Recruitment status | 50% of the recruitment completed |
| Primary outcome(s) | Feasibility and Safety Outcomes |
| Key secondary outcomes | Postural control - Mini-Balance Evaluation Systems Test (Mini-BEST-Test)  Gait - Functional Gait Assessment (FGA)  Cognition - Montreal Cognitive Scale (MoCA)  Quality of life - Brazilian version of the Short Form 36 (SF-36)  Mood- Geriatric Depression Scale (GDS-15)  Fear of falling - Falls Efficacy Scale (FES-I) |
