# Supplementary material for: Feasibility, safety, acceptability, and functional outcomes of playing Nintendo Wii Fit Plus™ for frail elderly: study protocol for a feasibility trial
Source: Pilot Feasibility Stud. 2017 Oct 24;3:41. doi: 10.1186/s40814-017-0184-1 (PMC5654058; doi:10.1186/s40814-017-0184-1)
Supplement: Supplementary file 2 — School of medicine of the University of Sao Paulo free and informed consent term. (DOCX 15 kb) [file 40814_2017_184_MOESM2_ESM.docx]

**Appendix A**

**SCHOOL OF MEDICINE OF THE UNIVERSITY OF SÃO PAULO**

**FREE AND INFORMED CONSENT TERM**

_______________________________________________________________________________

**IDENTIFICATION INFORMATION OF THE SUBJECT OF THE RESEARCH OR LEGAL ENTITY**

_______________________________________________________________________________

**1**. NAME: ...............................................................................................................................

IDENTITY DOCUMENT NUMBER: ........................................ SEX : .M □ F □

DATE OF BIRTH: ......../......../......

ADDRESS ..................................................................... Nº............ APARTMENT: ..............

NEIGHBORHOOD:.................................................................... CITY:.................................

ZIP CODE:..........................................TELEPHONE NUMBER: (.........).............................

**2**. ENTITY LEGAL...............................................................................................................

IDENTITY DOCUMENT NUMBER: ........................................ SEX : .M □ F □

DATE OF BIRTH: ......../......../......

ADDRESS ..................................................................... Nº............ APARTMENT: ..............

NEIGHBORHOOD:.................................................................... CITY:.................................

ZIP CODE:..........................................TELEPHONE NUMBER: (.........).............................

_________________________________________________________________________________

**RESEARCH DATA**

**1**. TITLE OF THE RESEARCH PROTOCOL: FEASIBILITY, SAFETY, ACCEPTABILITY AND FUNCTIONAL OUTCOMES OF PLAYING NINTENDO WII FIT PLUS FOR FRAIL ELDERLY

RESEARCHER: José Eduardo Pompeu

OFFICE/FUNCTION: Professor of the Department of Speech Therapy, Physiotherapy and Occupational Therapy, University of Sao Paulo.

INSCRIPTION REGIONAL COUNCIL: Crefito 3: 19.445-F

UNITY OF USP: Clinics Hospital of the Medical School at the University of São Paulo located in Dr. OvídioPires de Campos, 333 Cerqueira César 05403010 - São Paulo, SP – Brazil. Phones: (11) 26617813 Fax: (11) 26616888.

**2**.RESEARCH RISK ASSESSMENT:

MINIMUM RISK MEDIUM RISK □

LOW RISK X GREATER RISK □

**3**. RESEARCH DURATION: one year.

**FACULTY OF MEDICINE OF THE UNIVERSITY OF SAO PAULO**

Mr.(s) is being invited to participate in a research project named: “Feasibility, safety and functional outcomes of playing Nintendo Wii Fit Plus for frail elderly: a Randomised controlled feasibility trial” that aims to verify the feasibility, safety and possible adverse effects and effects of training with the Nintendo Wii Fit video game in elderly individuals with Frailty Syndrome.
 Mr. (s) will undergo an assessment of feasibility, acceptability, and safety through specific questions asked by a physical therapist, and balance, gait, memory, mood and quality of life assessments through specific tests. These assessments will occur at three moments: at the beginning, at the end, and 30 days after the end of the protocol.
 After the initial evaluation, you will participate in a draw to decide which of the two types of training will be involved in (control or experimental group). The experimental group will perform training sessions with the Nintendo Wii Fit Plus twice a week, lasting 50 minutes at the Clinics Hospital of the Medical School at the University of São Paulo located at 255, Doctor Ovídio Pires de Campos, Cerqueira César, São Paulo, SP – Brazil, Zip Code: 05403010. Telephones: (11) 26617813. The control group will be guided through verbal instructions and an illustrative booklet of guidelines that describes physical activity, its benefits and risks, and encourages the study participants to seek health units near their residence where free physical activities are offered.

Video game training will consist of five games involving exercises that move the head, arms, hands, legs, and feet. The trainings sessions will be demonstrated and supervised by a physical therapist.

Mr.(s) should be aware that during the training sessions you may experience some discomfort, such as dizziness, instability, and discomfort. Should any discomfort occur at any time in this research, Mr.(s) should notify the responsible physical therapist, who will take all the necessary steps to resolve these discomforts (from the activation of an emergency service to referral to a specific professional).
 The proposed training could contribute to improved balance, way of walking, breathing, and memory. In addition, if the results of this study confirm these benefits, the study may contribute to the improvement of the health of other elderly people through interactive video games.
 The principal investigator of the study is Professor José Eduardo Pompeu, who can be located at 51 Cipotânea Street, University City, ZIP CODE: 05360-000, telephone: 3091-8424 or e-mail: jepompeu@usp.br. Professor Pompeu will be on hand to answer any questions or clarify any questions at any time during the study. If you have any questions or concerns about the ethics of the research, Mr.(s) may contact the Research Ethics Committee of the Faculty of Medicine of the University of Sao Paulo, located at Dr. Arnaldo Avenue, number 251 - 21º floor - Cerqueira Cesar - Sao Paulo - SP, ZIP CODE: 01246-00001246-000. Telephone / Fax: 55 11 3893-4401 / 4407 or e-mail: cep.fm@usp.br.
 Mr.(s) is guaranteed the freedom to withdraw consent at any time and to stop participating in the study without any loss. The information obtained will be used only for scientific research purposes. Mr. (s) will be kept up to date on the partial results of the research, when in open studies, or results that are known to the researchers. There will be no personal expenses for the participant at any stage of the study, and there is no financial compensation related to their participation. If there is any additional expense, it will be absorbed by the research budget. The researchers agree to use the data and material collected for this research only.

I believe I have been sufficiently informed about the information I read or that was read to me describing the study: “Feasibility, safety and functional outcomes of playing Nintendo Wii Fit Plus for frail elderly: a randomized clinical trial”
 I discussed with Dr. (a) ______________________________________ about my decision to participate in this study. It was clear to me what the purposes of the study were, the procedures to be carried out, their discomforts and risks, the guarantees of confidentiality and permanent clarification. It is also clear that my participation is free of expenses and that I have guaranteed access to hospital treatment, when necessary. I voluntarily agree to participate in this study and may withdraw my consent at any time, before or during the research, without penalty, loss or loss of any benefit I may have acquired, or in my attendance at this Service.

_______________________________________________________________________

Signature of patient / legal representative - Date         /       /

_______________________________________________________________

Signature of the witness (For illiterate, semi-literate or hearing or visual impaired patients)– Date ___/___/___

I declare that I have obtained in an appropriate and voluntary manner the free and Informed Consent of this patient or legal representative for participation in this study.


______________________________________________________________

Prof. Dr. José Eduardo Pompeu; Phone: 3091-8424. - Date ___ / ___ / ___
